# Simulation of short-term pressure regulation during the tilt test in a coupled 3D–0D closed-loop model of the circulation

**DOI:** 10.1007/s10237-014-0645-x

**Published:** 2015-01-08

**Authors:** Kevin D. Lau, C. Alberto Figueroa

**Affiliations:** 1 Department of Biomedical Engineering, King’s College London, London, SE1 7EH UK; 2 Departments of Surgery and Biomedical Engineering, University of Michigan, Ann Arbor, MI 48109 USA

**Keywords:** Baroreflex, Blood flow, Transitional, Multi-scale, Predictive

## Abstract

Short-term fluctuations in arterial pressures arising from normal physiological function are buffered by a negative feedback system known as the arterial baroreflex. Initiated by altered biomechanical stretch in the vessel wall, the baroreflex coordinates a systemic response that alters heart rate, cardiac contractility and peripheral vessel vasoconstriction. In this work, a coupled 3D–0D formulation for the short-term pressure regulation of the systemic circulation is presented. Including the baroreflex feedback mechanisms, a patient-specific model of the large arteries is subjected to a simulated head up tilt test. Comparative simulations with and without baroreflex control highlight the critical role that the baroreflex has in regulating variations in pressures within the systemic circulation.

## Introduction

As part of the circulatory system, the systemic circulation functions to transport oxygenated blood throughout the human body via a network of branching and tapering elastic vessels. Driven by the pulsatile contraction of the heart, pressure and flow in this network vary from highly transient, large amplitude waveforms in the large vessels close to the heart, to relatively constant, small amplitude waveforms in the small vessels far from the heart. By transforming the nature of these waveforms, the systemic circulation provides a relatively constant supply of blood throughout the body. To maintain this supply of blood under a range of different physiological conditions, such as changes in posture, digestion, stress, trauma or exercise, the circulatory system is equipped with several regulatory feedback mechanisms. Affecting local and global properties such as individual vessel tone and heart rate, these feedback mechanisms enable the regulation of pressure and flow throughout the body.

**Fig. 1:**
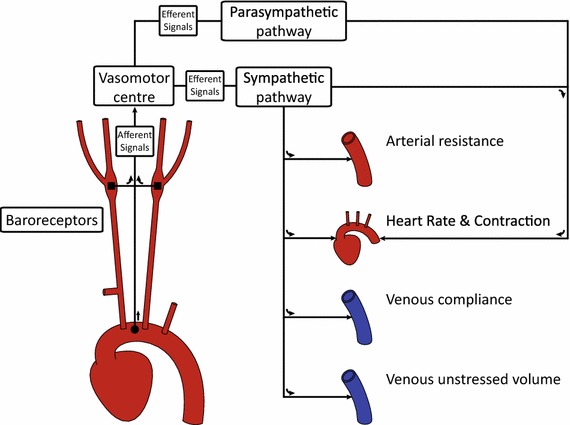
Schematic of the main components of the baroreflex. Here, the *filled square* and *filled circle* symbols refer to the location of the carotid and aortic baroreceptors, respectively

One such key regulatory mechanism is the arterial baroreflex—a negative feedback system that responds to short-term variations in pressure by altering the state of the systemic circulation in order to maintain pressure homoeostasis. The baroreflex can be broadly divided into three components: (1) the baroreceptors cells, (2) the vasomotor control center and (3) the sympathetic and parasympathetic nervous systems (see Fig. 1).

The baroreceptors are stretch-sensitive nerve cells located at the aortic arch and the carotid bifurcation. These cells detect changes in pressure by using the wall stretch as a direct surrogate. Connected to the vasomotor center of the brain via the afferent pathways, the baroreceptor cells modulate their nervous firing rate when the magnitude of stretch deviates from prior baseline values. Equipped with a memory of 1–2 days, these cells are specialized for the role of short-term pressure regulation (Guyton 1992).

The vasomotor center of the brain subconsciously interprets the afferent nervous activity of the baroreceptors. Altered afferent activity arising from variations in pressure generates efferent activity within the vasomotor center that is transmitted to different anatomical regions of the systemic circulation. This efferent activity travels through two different pathways, known as the sympathetic response and the parasympathetic response, which aim to restore blood pressure to baseline values.

The sympathetic and parasympathetic nervous systems innervate the heart and the peripheral vessels, thereby allowing control of heart rate, cardiac contractility and vessel vasoconstriction. Increased sympathetic activity results in vasoconstriction of the peripheral vessels, increased heart rate and increased cardiac contractility—factors which all have a restorative effect on blood pressure. Conversely, increased parasympathetic activity decreases heart rate and cardiac contractility, thereby reducing systemic blood pressure (Guyton 1992). Changes in sympathetic and parasympathetic activities occur simultaneously, e.g., an increase in sympathetic activity and a decrease in parasympathetic activity are effected when an increase in blood pressure is desired. Via the coordinated response of these activities, the baroreflex rapidly alters the hemodynamics throughout the systemic circulation, exerting global control of the pressure on a beat-by-beat basis. The ability to transiently alter pressure is clinically measured using an index referred to as the baroreflex sensitivity (La Rovere et al. 2008). Defined as the change in peak systolic pressure over successive beats, this sensitivity is a direct measure of the strength of the baroreflex response and has been shown to be an indicator of mortality in diseased states (La Rovere et al. 2001).

Clinical assessment of the baroreflex can be performed pharmacologically through hypotensive or hypertensive drug infusion, mechanically through compression via a neck collar apparatus or through controlled changes in posture—such as in the tilt test. Of the approaches listed, the tilt test is the least invasive, consisting of a controlled change in posture of the conscious patient from a supine (horizontal) to a upright (vertical) position (La Rovere et al. 2001). This change in posture induces a change in the pressure sensed by the baroreceptors located at the carotid bifurcation and the aortic arch that ultimately initiates the baroreflex response.

In this paper, we are interested in modeling the behavior of the baroreflex system using computational methods. In particular, we aim to reproduce the transient hemodynamics arising due to the short-term baroreflex regulation of blood pressure triggered by a virtual tilt test experiment.

Several mathematical models of the baroreflex have been proposed thus far, with each examining different aspects of this coupled system. So far these approaches have been mostly implemented using 0D (e.g. lumped parameter network) models, with one notable 3D contribution. To the best of the authors knowledge, there are currently no 1D models of the baroreflex in the literature.

Early 0D models of the baroreflex include those developed by Ottesen (1997) and Ursino (1998); these two models explored the long-term stability of the negative feedback mechanism and the effectiveness of the baroreflex in cases of blood loss (trauma), respectively. Other 0D models have examined the baroreflex under controlled scenarios, such as the tilt test in Heldt et al. (2002) where orientation dependent pressures were imposed in different anatomical regions. However, as 0D models lack a geometric component, the pressure gradient used to represent the effect of gravity was arbitrarily applied. More recently, Beard et al. (2013) examined the role of the baroreflex in combination with other regulatory pressure mechanisms, such as the renin-angiotensis system, to explore the effects of chronic baroreflex stimulation.

Although 0D models are numerically efficient and provide a simple framework in which the baroreflex response can be investigated, these models are unable to simulate features such as pulse wave propagation, complex local hemodynamics in stenoses and aneurysms, and most importantly for our purposes, gravity-induced gradients resulting in spatial variations in pressure. A combined 3D–0D modeling approach is required to properly represent these features. This approach captures complex hemodynamics in the 3D domain while modeling the baroreflex regulation in the 0D domain. This 0D domain is typically divided up into an upstream component (e.g., heart) and a downstream component (e.g., peripheral circulation).

A previous multi-domain 3D–0D model of the baroreflex was developed by Kim et al. (2010). There, a closed-loop model of the circulation was employed where a baroreflex response based on the model reported by Ottesen et al. (2004) was initiated by imposing arbitrary changes in the peripheral resistance of the systemic circulation. The controlled quantities were heart rate, cardiac contractility and peripheral arterial compliance.

In this work, we have used a 3D–0D approach whereby a less intrusive baroreflex trigger, given by the change in posture during the tilt test, was utilized. This approach enables the explicit control of the peripheral systemic resistance, one of the key factors effected by the baroreflex system. Furthermore, the tilt test naturally provides clinical data on heart rate, pressure and flow that can be used to inform and validate the computational model. Along with this improvement in the baroreflex triggering mechanism, we introduce the following novelties in our modeling framework:

- Improved design of the closed-loop lumped parameter model of the circulation: Here, the systemic peripheral vasculature is modeled using a multi-compartment network that represents the small arteries, arterioles, venules and veins. Furthermore, the flow entering the 0D domain from each outflow branch of the 3D domain is numerically handled to ensure global conservation of flow throughout the system.
- Improved ventriculo-arterial coupling: Coupling of the 0D left ventricle to the 3D domain includes a nonlinear left ventricular resistance to account for the flow rate dependent pressure losses that have been experimentally observed by Shroff et al. (1985).
- Improved baroreflex control: In this study, the model of the baroreflex regulates heart, cardiac contractility, arterial resistances, venous compliance and venous unstressed volume. The parameters defining the steady-state response of these hemodynamic variables have been fitted to physiological values reported in the literature.

The structure of this paper is as follows: Firstly, a detailed description of the multi-domain 3D–0D closed-loop model used to represent the systemic circulation is given in Sect. 2.1. This is followed by a characterization of the baroreflex response in Sect. 2.2. The implementation of the physical trigger used to stimulate the baroreflex is described in Sect. 2.3. The results of different numerical experiments, with and without baroreflex regulation, are presented in Sect. 3. Finally, the limitations of this study and future work are described in Sect. 4.

## Methods

### Closed-loop model of the systemic circulation

The model of the systemic circulation forms a closed loop that begins at the left ventricle and ends at the left atrium. Here, the pulmonary circulation has been neglected as the baroreflex predominantly alters the physiology of the systemic circulation. A circuit was constructed by coupling together the following 0D and 3D compartments: left ventricle (0D), large arteries (3D), small arteries (0D), arterioles (0D), venules (0D), veins (0D) and left atrium (0D). Hemodynamics in this closed-loop circuit were described using the coupled multi-domain approach originally presented in Vignon-Clementel et al. (2006). In the following sections, the 3D and 0D components of this circuit are described in further detail.

#### 3D model of the large arteries

**Fig. 2:**
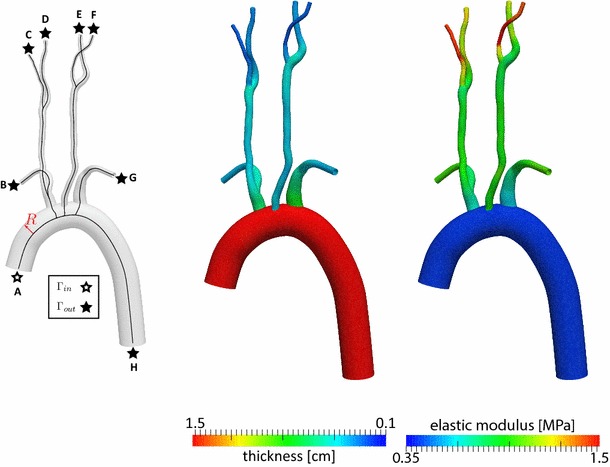
*Left* Model geometry with center lines and local radius $R$; center lines were calculated using the Vascular Modeling Toolkit http://www.vmtk.org/. *Center* Wall thickness, units in cm. *Right* Elastic wall modulus, units in MPa. Figure legend: *A* ascending aorta, *B* right subclavian, *C* right external carotid, *D* right internal carotid, *E* left internal carotid, *F* left external carotid , *G* left subclavian and *H* descending aorta

A patient-specific CAD model of the large arteries of the chest and neck was derived from a previous study (Coogan et al. 2013). This model contains the following vessels: ascending and descending aorta, left and right subclavians, left and right internal carotids, and left and right external carotids (see Fig. 2). The extent of the model is therefore sufficient to capture the location of the two sets of baroreceptor cells at the aortic arch and the carotid bifurcations. The CAD model was discretized using a combination of global mesh size ($h$ $=$ 0.1 cm) and curvature-based refinement. This produced a 1,795,001 tetrahedral element mesh. A pulsatile simulation in pre-tilt conditions was then run, followed by a field-based anisotropic mesh refinement as described in Sahni et al. (2006). This procedure generated a 973,882 tetrahedral element mesh, with identical results for flow and pressure waveforms as the initial mesh. The field-adapted mesh was then used in the tilt test simulations.

The physics of blood in the 3D domain has been described by the incompressible Navier–Stokes equations for a Newtonian fluid coupled to the (linear) elastodynamics equations for the vessel wall characterized via a thin membrane. This coupled system has been solved using a 3D stabilized finite-element approach; details of this approach have been described elsewhere (Whiting and Jansen 2001; Vignon-Clementel et al. 2006; Figueroa et al. 2006). Briefly, the weak form of the coupled system is given by the following variational equation1where p and $\mathbf {v}$ are the blood pressure and velocity, respectively; $\varOmega$ is the blood flow domain; $q$ and $\mathbf {w}$ are the test functions for the mass and momentum balance, respectively; $\rho$ is the blood density;  is the viscous stress tensor for a Newtonian fluid where $\mu$ is the dynamic viscosity; $\mathbf {f}$ is body force per unit volume; $\varGamma _{g}$ is a Dirichlet boundary where the test function $\mathbf {w}$ vanishes; $\varGamma _{h}$ is the interface where $\mathbf {t}^{h}$ is the traction defined by the 3D–0D coupling; $\varGamma _{t}$ is the interface with the vessel wall where a traction $\mathbf {t}^{t}$ resulting from the fluid–solid interaction is defined; and $\mathbf {n}$ is the normal vector on each boundary. The traction $\mathbf {t}^{h}$ on $\varGamma _{h}$ is defined by the choice of reduced-order model for the proximal and distal regions of the systemic circulation, viz.  (Kim et al. 2010). The boundary $\varGamma _{h}$ is subdivided into inflow $\varGamma _{\mathrm{in}}$ and outflow $\varGamma _{\mathrm{out}}$ surfaces, such that $\overline{\varGamma _{\mathrm{in}} \cup \varGamma _{\mathrm{out}}} = \varGamma _{h}$ and $\varGamma _{\mathrm{in}} \cap \varGamma _{\mathrm{out}} = \varnothing$. The inflow surface $\varGamma _{\mathrm{in}}$ switches between a Neumann boundary during systole (aortic valve open) and a Dirichlet boundary during diastole (aortic valve closed), see Sect. 2.1.2. Neumann boundaries have been previously shown to require numerical stabilization during periods of flow reversal (Moghadam et al. 2011). In this work, the stabilization approach detailed by Hughes and Wells (2005) was adopted, where the resulting deficit between the total flux traction and the 0D pressure was corrected following a similar approach to that reported in Ismail et al. (2013).

**Fig. 3:**
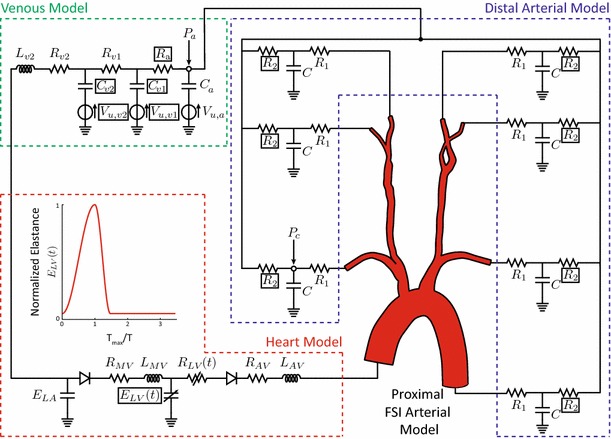
Model of the systemic circulation with both 3D and 0D components. Here, the pressures compliance chamber and arterioles pressures, $P_{\mathrm{c}}$ and $P_{\mathrm{a}}$, respectively, are highlighted with the *open circle* symbols; the *boxed variables* denote that these are controlled quantities

On $\varGamma _{t}$, an incompressible, linearly elastic membrane (Figueroa et al. 2006) of thickness $\zeta$, defined as 10 % of the local vessel radius $R$, was utilized. An isotropic elastic modulus $E$ was defined regionally as

2 $$\begin{aligned} E = E^{*} \frac{3 \rho R c^{2}}{2 \zeta }, \end{aligned}$$

where $c$ is the local pulse wave velocity and $E^{*} = 2$ is a scaling factor. Here, the local pulse wave velocity $c$ was calculated using an empirical formula reported by Reymond et al. (2009),

3 $$\begin{aligned} c \left( R \right) \approx \frac{13.3}{(2R)^{0.3}}. \end{aligned}$$

The distribution of mechanical properties (thickness and stiffness) of the 3D anatomical model is illustrated in Fig. 2.

#### 0D model of the left heart

The left heart model is divided into a series of 0D components representing the atrial and ventricular compartments and the valves separating them (Fig. 3). The pulsatile contraction and relaxation of the left ventricle was modelled using an elastance function $E_{\mathrm{LV}}$, defined here as the time-varying ratio of the ventricular pressure $P_{\mathrm{LV}}$ to the difference between the ventricular volume $V_{\mathrm{LV}}$ and the unstressed ventricular volume $V_{\mathrm{u},\mathrm{LV}}$,

4 $$\begin{aligned} E_{\mathrm{LV}}(t) = \frac{P_{\mathrm{LV}}(t)}{V_{\mathrm{LV}}(t) - V_{\mathrm{u},\mathrm{LV}}}. \end{aligned}$$

Experimental studies under differing heart rates and contractile states, in both normal and diseased hearts, have shown that the elastance function can be reduced to a universal non-dimensional curve (Suga and Sagawa 1974). Normalized by its maximal value $E_{\mathrm{max}}$ and the corresponding time to this maximum $T_{\mathrm{max}}$, this non-dimensional function represents the pulsatile behavior of the ventricle under a range of different conditions. Here, we have adopted the analytical function reported by Pope et al. (2009) (Fig. 3) to characterize the normalized elastance function.

The mitral and aortic valves were modeled via diodes that permit flow only in the forward direction. The opening of these valves is triggered by a negative pressure gradient in the direction of flow; once opened, the valve remains open until retrograde flow is detected—regardless of the pressure gradient. The closure of the aortic valve disconnects the 3D domain from the heart model. At this point, a zero velocity Dirichlet boundary condition is imposed on the surface $\varGamma _{\mathrm{in}}$. When the aortic valve reopens the boundary, $\varGamma _{\mathrm{in}}$ is coupled to the heart model via a pressure Neumann boundary condition described by the following set of ODEs

5 $$\begin{aligned} \begin{bmatrix} L_{\mathrm{AV}}\frac{\mathrm{d} Q_{\mathrm{AV}}}{\mathrm{d}t} + R_{\mathrm{AV}} Q_{\mathrm{AV}} + R_{\mathrm{LV}} Q_{\mathrm{AV}} \\ \frac{\mathrm{d}V_{\mathrm{LV}}}{\mathrm{d}t} \end{bmatrix} = \begin{bmatrix} P_{\mathrm{LV}} - P_{\mathrm{AV}} \\ -Q_{\mathrm{AV}} \end{bmatrix}, \end{aligned}$$

where $P_{\mathrm{AV}}$ and $Q_{\mathrm{AV}}$ are the average pressure and flow on $\varGamma _{\mathrm{in}}$, respectively; $R_{\mathrm{AV}}$ is the aortic resistance; $L_{\mathrm{AV}}$ is the aortic inductance; and $R_{\mathrm{LV}}$ is the internal nonlinear ventricular resistance defined as a function of the current ventricular pressure and the constant $k_{\mathrm{LV}}$ (see Fig. 3)

6 $$\begin{aligned} R_{\mathrm{LV}}(t) = k_{\mathrm{LV}} P_{\mathrm{LV}}(t). \end{aligned}$$

This internal resistance has been shown to directly affect the shape of the aortic flow waveform (Shroff et al. 1985). Included in previous 1D models (Reymond et al. 2009), here this component has been implemented for the first time in a 3D setting. Writing the left ventricular pressure $P_{\mathrm{LV}}$ as a function of elastance and ventricular volume, the coupled ODEs in Eq. 5 can be further simplified. The resulting nonlinear equations are solved numerically using a backward Euler scheme and a Newton–Raphson method.

The left atrium enables the refilling of the left ventricle during the relaxation phase of the cardiac cycle. Modeled as a passive component with a constant elastance $E_{\mathrm{LA}}$, the pressure in the left atrium is defined as $P_{\mathrm{LA}}(t) = E_{\mathrm{LA}} ( V_{\mathrm{LA}}(t) - V_{\mathrm{u},\mathrm{LA}} )$, where $V_{\mathrm{LA}}$ and $V_{\mathrm{u},\mathrm{LA}}$ represent the volume and unstressed volume of the atrium, respectively. The left atrium equations are solved simultaneously with the equations characterizing the venous and distal arterial components of the circuit, see Sect. 2.1.3 below. The values of all the heart model parameters are detailed in Table 1.

**Table 1:** Heart model parameters

Left ventricle					
$E_{\mathrm{min}}$	$E_{\mathrm{max}}$	$T_{\mathrm{max}}$	$T_{\mathrm{relax}}$	$k_{\mathrm{LV}}$	$V_{\mathrm{u},\mathrm{LV}}$
$4.102 \times 10^{1}$	$3.000 \times 10^{3}$	$4 \times 10^{-1}$	$2 \times 10^{-1}$	$5 \times 10^{-4}$	0.

Left atrium		Mitral valve		Aortic valve	
$E_{\mathrm{LA}}$	$V_{\mathrm{u},\mathrm{LA}}$	$R_{\mathrm{MV}}$	$L_{\mathrm{MV}}$	$R_{\mathrm{av}}$	$L_{\mathrm{av}}$
$1.333 \times 10^{2}$	0.	$1.187 \times 10^{-1}$	$6.667 \times 10^{-1}$	$1.000 \times 10^{-1}$	$1.000 \times 10^{-1}$

The units of $E_{\mathrm{max}}$, $E_{\mathrm{min}}$ and $E_{\mathrm{la}}$ are dynes $\hbox {cm}^{-5}$. The units of $k_{\mathrm{LV}}$ are $\hbox {s} \, \hbox {cm}^{-3}$. The units of the valve resistances $R_{\mathrm{MV}}$ and $R_{\mathrm{AV}}$ are dynes $\hbox {s} \, \hbox {cm}^{-5}$. The units of the valve inductances $L_{\mathrm{MV}}$ and $L_{\mathrm{AV}}$ are dynes $\hbox {s}^{2} \, \hbox {cm}^{-5}$

#### 0D model of the peripheral systemic vasculature

At each outlet branch of the 3D domain, the corresponding surface $\varGamma _{\mathrm{out}}$ is coupled to a 3-element Windkessel model that represents the small arteries immediately downstream (Fig. 3). The pressure $P_{i}$ and flow $Q_{i}$ relationship on the $i$th branch are described by the following equations

7 $$\begin{aligned} \begin{bmatrix} P_{i} - P_{c,i} \\ C_{i} \frac{\hbox {d}P_{c,i}}{\hbox {d}t} \end{bmatrix} = \begin{bmatrix} R_{1,i} Q_{i} \\ Q_{i} - \frac{P_{c,i} - P_{a}}{R_{2,i}} \end{bmatrix} , \end{aligned}$$

where $P_{c,i}$ is the pressure at the compliance chamber; $C_{i}$ is the compliance; $R_{1,i}$ and $R_{2,i}$ are proximal and distal resistances, respectively; and $P_{\mathrm{a}}$ is the arteriole pressure (see Fig. 3). Here, the value of the proximal resistance $R_{1,i}$ is matched to the characteristic outlet impedance of the vessel using the equation

8 $$\begin{aligned} R_{1,i} = \frac{\rho c}{A_{i}} , \end{aligned}$$

where $A_{i}$ is the area of the outlet surface. Following the iterative approach described in Xiao et al. (2014), the Windkessel parameters were tuned to produce a division of flow proportional to the area of each outlet, and a mean aortic pressure of $120$ mmHg, with a pulse pressure of $30$ mmHg. These parameters are listed in Table 2.

**Table 2:** Resistances and compliances of the small arteries

Vessel	$R_{1}$	$C$	$R_{2}$
Descending aorta	$1.052 \times 10^{2}$	$9.995 \times 10^{-4}$	$1.650 \times 10^{3}$
Right subclavian	$4.506 \times 10^{3}$	$3.963 \times 10^{-5}$	$4.162 \times 10^{4}$
Left subclavian	$4.922 \times 10^{3}$	$3.673 \times 10^{-5}$	$4.491 \times 10^{4}$
Right internal carotid	$7.868 \times 10^{3}$	$2.454 \times 10^{-5}$	$6.722 \times 10^{4}$
Left internal carotid	$8.481 \times 10^{3}$	$2.310 \times 10^{-5}$	$7.141 \times 10^{4}$
Right external carotid	$1.898 \times 10^{4}$	$1.161 \times 10^{-5}$	$1.421 \times 10^{5}$
Left external carotid	$1.739 \times 10^{4}$	$1.251 \times 10^{-5}$	$1.318 \times 10^{5}$

The units of the resistances $R$ are dynes $\hbox {s} \, \hbox {cm}^{-5}$. The units of the inductances L are dynes $\hbox {s}^{2} \, \hbox {cm}^{-5}$. The units of the compliance $C$ are $\hbox {cm}^{5} \, \hbox {dynes}^{-1}$. The units of the unstressed volumes $V_\mathrm{u}$ are $\hbox {cm}^{3}$

The termination of each 3-element Windkessel model is coupled to a lumped parameter circuit that represents the arterioles, venules and veins (Fig. 3). The final venous compartment connects directly to the left atrium, bypassing the pulmonary circulation, thus closing the loop formed by this systemic circuit. In this approach, the flow from each branch is explicitly gathered and passed on to the arterioles and venous compartments, thus enforcing the proper continuity of flow within the system. This circuit is derived from that originally reported by Ottesen et al. (2004). The parameters for the arterioles, venules and venous compartments are detailed in Table 3.

**Table 3:** Resistances, compliances and unstressed volumes of the arterioles, venules and venous compartments

Compartment	$R$	$L$	$C$	$V_{u}$
Arterioles	$8.893 \,{\times }\, 10^{2}$	–	$1.400 \,{\times }\, 10^{-3}$	$4.010 \,{\times }\, 10^{2}$
Venules	$2.973 \,{\times }\, 10^{1}$	–	$9.900 \,{\times }\, 10^{-3}$	$5.960 \,{\times }\, 10^{2}$
Venous	$3.560 \,{\times }\, 10^{1}$	$6.670 \,{\times }\, 10^{-2}$	$5.540 \,{\times }\, 10^{-2}$	$1.938 \,{\times }\, 10^{3}$

The units of the resistances $R$ are dynes $\hbox {s} \, \hbox {cm}^{-5}$. The units of the inductances $L$ are dynes $\hbox {s}^{2} \, \hbox {cm}^{-5}$. The units of the compliance $C$ are $\hbox {cm}^{5} \, \hbox {dynes}^{-1}$. The units of the unstressed volumes $V_\mathrm{u}$ are $\hbox {cm}^{3}$

The 3-element Windkessels, arterioles, venules, veins and left atrial compartments result in a system of first-order ODEs. These equations are all solved together using a backward Euler scheme, resulting in an algebraic system of the form:

9 $$\begin{aligned} \mathbf {A} \mathbf {x}^{n+1} = \mathbf {B} \mathbf {x}^{n} + \mathbf {C} \mathbf {q}^{n+1} + \mathbf {D} , \end{aligned}$$

When the mitral valve is open, these equations are expanded to also include the left ventricular compartment. Here, the vector $\mathbf {x}^{n+1}$ represents the internal states of the circuit (pressures, volumes and flows), and $\mathbf {q}^{n+1}$ is the vector of outlet flows in the 3D domain at the discrete time $t = (n+1) \Delta t$. The matrices $\mathbf {A}, \, \mathbf {B}, \, \mathbf {C}$ and $\mathbf {D}$ contain the resistors, inductors, capacitors and source components of the lumped parameter circuit representing the 0D closed-loop circuit. This system of equations (Eq. 9) is solved via LU factorization.

### Baroreflex response

The baroreceptor cells modify their firing rate (i.e., afferent activity) in response to deviations in pressure. Here, this activity was modeled using the index $\delta$ proposed in Ottesen (1997) which is defined as the ratio of the current cardiac cycle average pressure $\overline{p}$ to some predefined (i.e., preferred) pressure ${p}_{\mathrm{target}}$, viz.

10 $$\begin{aligned} \delta = \frac{\overline{p}}{p_{\mathrm{target}}} . \end{aligned}$$

The value of $p_{\mathrm{target}}$ was defined for each carotid vessel as the pre-tilt value of $\overline{p}$. Increases or decreases in blood pressure result in larger or smaller values of the index $\delta$. Given the three spatial locations of the baroreceptor cells (left and right carotid bifurcation and aortic arch), there are potentially different deviations in baroreceptor afferent activity. In this work, the pressure $\overline{p}$ was calculated on each of the internal and external carotid outflow surfaces (faces C, D, E, F in Fig. 2). For the sake of simplicity, it was assumed that the largest deviation $\delta$ drives the overall response of the baroreflex system.

Based on the experimental reports of Korner (1971), Ottesen and colleagues proposed the following sigmoidal relationships for the efferent responses:

11 $$\begin{aligned}&n_{s}(\delta )=\frac{1}{1 + \delta ^{+\nu }} \end{aligned}$$

12 $$\begin{aligned}&n_{p}(\delta )=\frac{1}{1 + \delta ^{-\nu }}, \end{aligned}$$

where $n_{\mathrm{s}}$ is the normalized sympathetic activity, $n_{\mathrm{p}}$ is the normalized parasympathetic activity, and $\nu = 5$ is a steepness parameter. These functional relationships describe the observed efferent response whereby increases in pressure simultaneously increase and decrease sympathetic and parasympathetic activity, respectively; conversely decreases in pressure cause a decrease in sympathetic activity and an increase in parasympathetic activity. With further increases (or decreases) in pressure, the nervous activity of each system continues increasing or decreasing until it saturates at either 1 or 0.

**Fig. 4:**
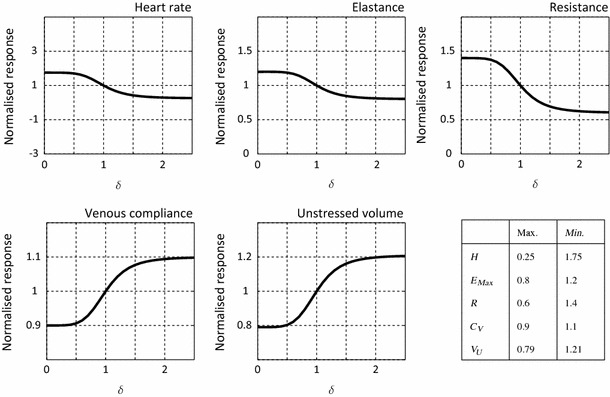
Normalized control response for the control variables. Labels: $\delta$—afferent activity, *H*—heart rate, $E_{Max}$—maximum elastance, *R*—arterial resistance, $C_{V}$—venous compliance, $V_{\mathrm{U}}$—unstressed volume

The nervous activity of the sympathetic and parasympathetic systems controls the properties of the systemic circulation, namely the heart period $T$, maximum ventricular elastance $E_{\mathrm{Max}}$, peripheral arterial resistance $R_{2,i}$ and $R_{\mathrm{a}}$, venous compliance $C_{\mathrm{v}1}$ and $C_{\mathrm{v}2}$, and venous unstressed volume $V_{\mathrm{u},\mathrm{v}1}$ and $V_{\mathrm{u},\mathrm{v}2}$. Ottesen et al. (2004) modeled the change in each of these variables using the following ODE

13 $$\begin{aligned} \tau _{i} \frac{\mathrm{d}x_{i}}{\hbox {d}t} + x_{i} = \alpha _{i} n_{s} ( \delta ) - \beta _{i} n_{p} ( \delta ) + \gamma _{i} , \end{aligned}$$

where the subscript $i$ represents the $i$th normalized controlled property $x_{i}$, $\tau _{i}$ is its characteristic time constant, and $\alpha _{i}$, $\beta _{i}$ and $\gamma _{i}$ are the corresponding control gain parameters defining the steady-state response. Table 4 lists the numerical values of $\tau _{i}$, $\alpha _{i}$, $\beta _{i}$ and $\gamma _{i}$ that reproduce the steady-state response of the various controlled properties. As illustrated in Fig. 4, a decrease in pressure results in an increase in heart rate, maximum elastance and arterial resistance, with a simultaneous decrease in venous compliance and unstressed volume. Conversely, an increase in pressure results in response in which heart rate, maximum elastance and arterial resistance all decrease, while venous compliance and unstressed volume increase. Of note, while the sympathetic activity $n_{s}$ affects all controlled properties (i.e., $\alpha _i \ne 0 \,\, \forall i$), the parasympathetic activity was assumed to affect only the heart rate (i.e., $\beta _i = 0, \,\, i=2,3,4,5$).

**Table 4:** Control ODE parameters

$i$	Symbol	$\tau _{i}$	$\alpha _{i}$	$\beta _{i}$	$\gamma _{i}$
1	$H$	3	1.75	$-$0.25	0.00
2	$E_\mathrm{Max}$	3	0.40	0.00	0.80
3	$R$	3	0.80	0.00	0.60
4	$C_{V}$	30	$-$0.20	0.00	1.10
5	$V_{U}$	30	$-$0.42	0.00	1.21

Variables: *H* heart rate, $E_{Max}$ maximum elastance, *R* arterial resistance, $C_{V}$ venous compliance and $V_{\mathrm{U}}$ venous unstressed volume. Units of $\tau$ are $\hbox {s}^{-1}$ and $\alpha , \, \beta$ and $\gamma$ are dimensionless

### Tilt test stimulation of the baroreflex response

The tilt test is a medical procedure used to diagnose malfunctions in the autonomic nervous system. In this procedure, the patient lies flat on a special table and heart rate activity and blood pressure are monitored. The orientation of the table is then altered from a horizontal to a vertical configuration over the course of a few seconds, such that a gravity-induced change in pressure triggers the baroreflex response. Tilt test hemodynamics have been previously studied in a 0D setting (Heldt et al. 2002). In this work, we present the first 3D investigation of hemodynamical changes during the tilt test. Rather than physically rotating the 3D computational domain, the change in posture was simulated by altering the orientation of gravity in the domain via a time-varying body force per unit volume $\mathbf {f} = \rho \, \mathbf {g}(t)$. Here, the initial gravity vector $\mathbf {g}_{0} = [0 \,\,\,\, 0 \, -g]^{T}$ is oriented such that the patient begins in a supine (horizontal) position, with the $x$-coordinate running from the feet to the head and the $z$-coordinate running from the back to the chest of the patient. Then, a rotation $\theta (t)$ around the $y$-axis is imposed over the course of 5 s. The magnitude of the gravity vector has been taken as $g=9.810 \times 10^{2} \, \hbox {cm} \, \hbox {s}^{-2}$. Considering this, the time-varying gravity vector is defined as:

14 $$\begin{aligned} \mathbf {g}(t) = \left[ \begin{array}{c@{\quad }c@{\quad }c} \mathrm{\cos }(\theta (t)) &{} 0 &{} -\mathrm{\sin }(\theta (t)) \\ 0 &{} 1 &{} 0 \\ \mathrm{\sin }(\theta (t)) &{} 0 &{} \mathrm{\cos }(\theta (t)) \end{array} \right] \left[ \begin{array}{c} 0 \\ 0 \\ -g \end{array} \right] . \end{aligned}$$

## Results

To better understand the hemodynamic alterations introduced by our baroreflex model in response to changes in posture, two different simulations were performed—with and without the baroreflex response activated, herein referred to as the control and no-control cases. Each simulation consists of a total of 25 s of physical time, subdivided into three stages: (1) 10 s in a supine (horizontal) position, (2) 5 s of rotation from the supine to an upright position and (3) 10 s in a fixed upright position.

Simulations were performed in our custom parallel blood flow solver *CRIMSON* on 128 processors on a SGI Altix UV. Total wall time for each simulation was 96 h.

### Hemodynamics of tilt test: control and no-control cases

**Fig. 5:**
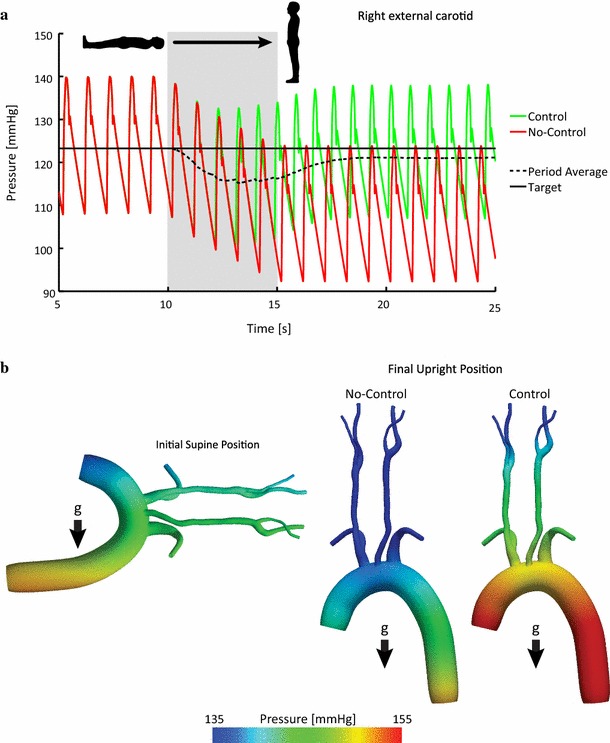
Effect of the baroreflex upon pressure in the large arteries. **a** Pressure history of the right external carotid. **b** Pressure distribution at peak systole pre-tilt and post-tilt, for both the no-control and control cases

Figure 5a presents the time history of pressure at the right external carotid for both cases, control and no-control. During the supine stage of the tilt test $(t<10 \,\hbox {s})$, identical periodic pressure waveforms are obtained in both simulations. At $t = 10 \, \hbox {s}$, the rotation of the table begins and pressure decreases rapidly in both cases. In the no-control simulation, the pressure continues to fall until the end of the rotation, whereby a new periodic state is achieved. In the control case, however, the error signal given by the difference between the (current) cardiac cycle average pressure (dashed line) and the target average pressure (solid line) triggers the response of the baroreflex. The ensuing action of the efferent pathways causes a rapid increase in pressure, resulting in significant differences between the control and no-control cases for $t>12 \,\hbox {s}$. Once the upright position is reached and maintained $(t>15 \,\hbox {s})$, a new periodic state pressure is developed, with an average pressure that is significantly closer to the original target value than the pressure observed on the no-control case.

Figure 5b illustrates the 3D distribution of pressure at peak systole in the supine and upright positions for both cases. Significant differences are observed between the control and no-control cases at the end of the upright stage of the test. In the no-control case, pressures in the carotid and subclavian vessels are significantly lower than in the initial supine position, however this difference is less prominent in the ascending and descending aorta. Conversely, in the control case, the efferent effect of the baroreflex resulted in increased pressures throughout the 3D domain. Pressures in the carotids exhibit values comparable to those found in the initial supine position while the aorta now presents with larger values than in the supine configuration.

In all cases, the effect of the gravity-induced pressure gradient can be clearly observed. The pressure differential across the model is more apparent in the upright configurations, as the longer dimension of the anatomy is aligned with the orientation of the gravity vector $\mathbf {g}$.

**Fig. 6:**
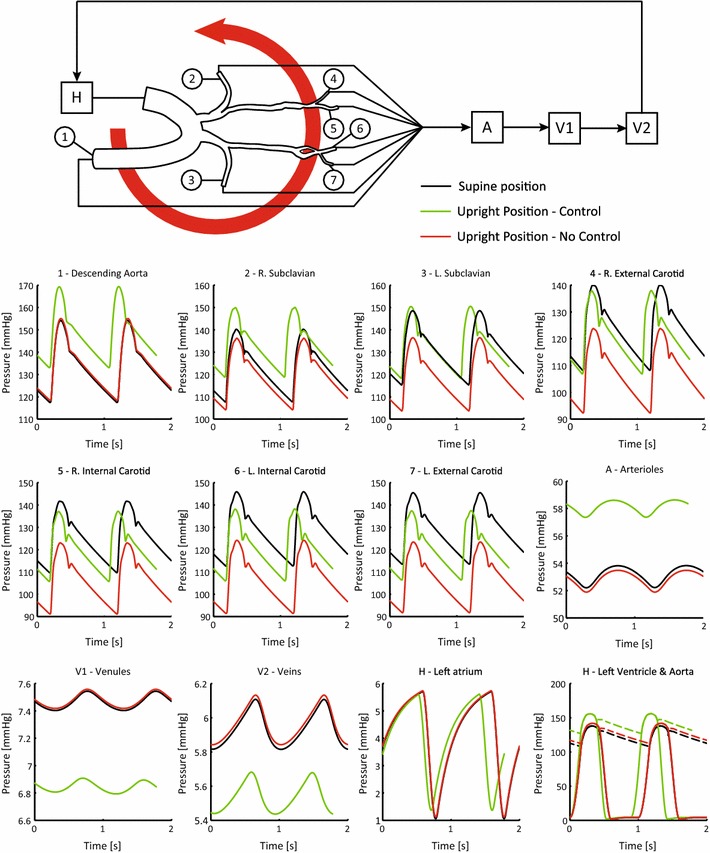
Periodic pressures in the closed-loop model in the initial supine and upright positions in both the no-control and control cases

A summary of the pressure waveforms throughout the closed-loop circuit is presented in Fig. 6. Pressure in the final two cycles of the supine position prior to the rotation of the table is compared with the pressure in the final two cycles of the simulation for both no-control and control cases. For the sake of clarity, all pressure waveforms have been aligned using the same reference point in time given by the start of the two cardiac cycles in each of the presented cases.

In all upper branch vessels (subclavians and carotids) and in the arterioles, the pressures in the no-control case are reduced relative to their initial supine values with typical reductions on the order of  10 mmHg. This is due to the pressure gradient created by the change in position and the lack of control. In the control case, however, pressures are all increased relative to the no-control simulation, with values close to or greater than the initial supine values.

In the venules and veins, changes in pressure exhibit a different pattern than that observed in the arterial vessels. Here, pressures remained virtually unaffected in the no-control case and decreased in the control case. The differences in pressure are significantly smaller than those observed in the arterial vessels, with maximum change of $<$1 mmHg.

Left atrial pressures remained relatively constant between supine and upright positions for both cases. The situation is different in the left ventricle, where slight pressure increases in the no-control case and significant pressure increases in the control case are observed. This increase in left ventricular pressure reflects the larger workload that the heart must generate to overcome the gravity-induced pressure gradient when the baroreflex response is turned on.

**Fig. 7:**
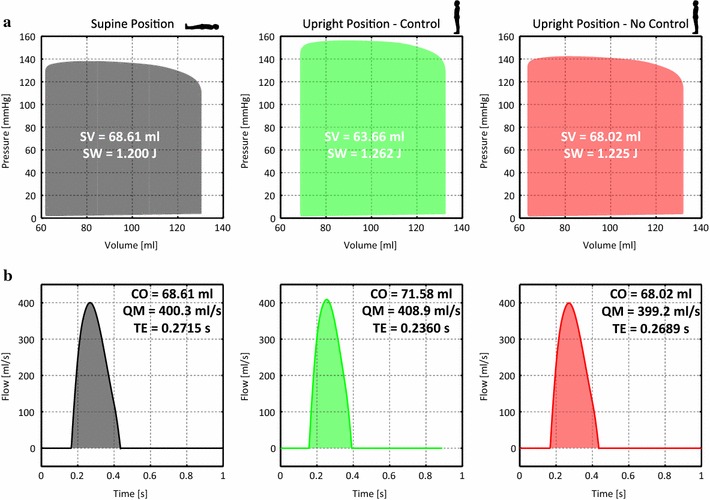
Ventricular pressure–volume loops and aortic flow waveforms in the initial supine and upright positions in both the no-control and control cases. Labels: *SV* stroke volume, *SW* stroke work, *CO* cardiac output, *QM* maximum flow rate, *TE* ejection time

Figure 7a shows the left ventricular pressure–volume loops for the supine, upright control and upright no-control cases. Values of stroke volume (in ml) and stroke work (in Joules) are provided in each plot. The upright no-control case exhibits similar values to those observed in the supine case. The upright control case, however, shows a larger stroke work (5 % increase) and smaller stroke volume (7 % decrease) relative to the supine case. This is once again a reflection of the additional work that the heart performs to overcome the effect of gravity when the baroreflex is turned on.

Figure 7b shows the aortic flow waveforms for the supine and upright positions, control and no-control. Numerical values of cardiac output (in ml/s), maximum flow rate (in ml/s) and ejection time (in seconds) are provided in each plot. While the waveforms have similar profiles in all cases, differences are observed in maximum flow rates and ejection times. The control case presents a slightly faster heart rate and larger cardiac output.

Table 5 summarizes the values of mean flows to each branch in the 3D model for the supine, upright control and upright no-control cases.

**Table 5:** Period average flows in the arterial vessels in supine $Q_{S}$, upright control $Q_{C}$ and upright no-control $Q_{N}$ cases

Vessel	$Q_{S}$	$Q_{C}$	$Q_{N}$
Left subclavian	2.073	1.884	1.764
Right subclavian	2.017	2.047	1.917
Left internal carotid	1.248	$9.815 \times 10^{-1}$	$8.944 \times 10^{-1}$
Right internal carotid	1.272	1.038	$9.453 \times 10^{-1}$
Left external carotid	$6.725 \times 10^{-1}$	$5.261 \times 10^{-1}$	$4.794 \times 10^{-1}$
Right external carotid	$5.802 \times 10^{-1}$	$4.924 \times 10^{-1}$	$4.492 \times 10^{-1}$
Descending aorta	$6.073 \times 10^{1}$	$6.419 \times 10^{1}$	$6.157 \times 10^{1}$

The units of flows are $\hbox {cm}^{3} \, \hbox {s}^{-1}$

**Fig. 8:**
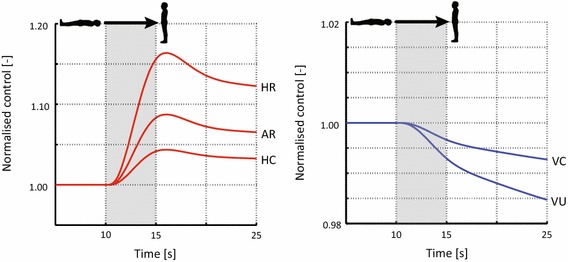
Normalized response of the heart rate (*HR*), maximum elastance (*HC*), arterial resistance (*AR*), venous compliance (*VC*) and venous unstressed volume (*VU*) in response to head up tilt. Here, the rotation portion of the tilt test occurs from $t = 10 \, \hbox {s}$ to $t = 15 \, \hbox {s}$

Figure 8 illustrates the time history of the normalized controlled variables in the closed-loop model. Triggered by the tilt test, the efferent response of the baroreflex model rapidly increases heart rate, maximum elastance and arterial resistance. These variables achieve their maximal values shortly after reaching the upright position at $t = 15$ s, eventually decreasing in value and converging to a new steady state at the end of the simulation. Conversely, the venous compliance and venous unstressed volume decrease in value during the rotation stage of the tilt test. Due to the differences in the dynamic response of these variables, a steady state is not fully achieved by the end of the simulation.

## Discussion

The baroreceptors are stretch-sensitive nerve cells located at the carotid bifurcation and aortic arch. Variations in pressure result in changes in stretch, and subsequent afferent activity between the baroreceptor cells and the central nervous system. In this work, a change in pressure was induced in the vascular model by virtually altering its orientation with respect to a given gravity field to simulate a clinical procedure known as the tilt test. As a result of this rotation, the upper branch vessels (subclavians and carotids) experience the largest change in elevation and therefore the greatest drop in pressure, as shown in Figs. 5 and 6.

### Baroreflex effect on the controlled variables

The efferent pathways of the baroreflex alter the heart rate, maximum elastance, arterial resistance, venous compliance and venous unstressed volume with the overall goal of regulating pressure in the systemic circulation (Fig. 8).

In the adopted model of the baroreflex (Ottesen et al. 2004), the efferent response is triggered by an error signal defined by the difference in the current cycle average carotid pressure relative to a target pressure. Due to differences in the characteristic time constants $\tau _{i}$ defining the speed of the response for the controlled variables (see Table 4), variations in heart rate, maximum elastance and arterial resistance occur significantly faster than variations in venous compliance and unstressed volumes. Peak values for the heart rate, maximum elastance and arterial resistance are observed at $t \approx 16 \, \hbox {s}$ (1 s after the initiation of the tilt test rotation). The strongest efferent response is observed in the heart rate, which has a maximum increase of ${\sim }$15 %. Conversely, the maximum variation in the venous compliance and unstressed venous volume is obtained at the end of the numerical experiment $(t = 25 \, \hbox {s})$. At this time, these variables have not yet attained a new steady state. Here, the venous compliance shows the weakest change during the tilt test, with a maximum decrease of just ${\sim }{-}$1 %.

### Baroreflex effect on the hemodynamics of the systemic circulation

Figure 5 illustrates the rapid response of the baroreflex on the right external carotid pressure. At $t \approx 11 \, \hbox {s}$ an increase in systolic pressure and a decrease in the period length are apparent in the control case relative to the no-control case. In the control case, carotid pressures continue to increase on a beat-by-beat basis, increasing the current cardiac cycle average pressure until a new steady-state value is reached at $t \approx 18 \, \hbox {s}$. Figure 6 shows the recovery of pressure in all of the carotid vessels, outlet numbers 4–7. This recovery occurs without exceeding the initial supine values. Of the four carotid vessels, the right external carotid (outlet number 4) recovers the most pressure, with the left external carotid (outlet number 7) recovering the least. In the descending aorta and left and right subclavian arteries, outlet numbers 1–3, the effect of the baroreflex enables these vessels to surpass their initial supine values. The effects of the baroreflex on pressure are also apparent in the distal compartments of the circuit. By increasing the resistance in the arterioles, the pressure is increased relative to the initial supine values; conversely, decreases in venous compliances and unstressed volumes have the opposing effect, decreasing pressure in the venules and veins.

The baroreflex also has an effect on the flows in the systemic circulation. Figure 7 illustrates the aortic flow waveforms for each of the different situations considered. Ejection time and maximum flow rate in the upright no-control case are virtually identical to those in the initial supine case ($2.600 \times 10^{-3} \, \hbox {s}$ difference and a ${\sim }$0.3 % drop, respectively).

In the upright control case, the perceived afterload change due to gravity is further increased by the gain in distal vasculature resistance. However, in this case, the increase in maximum elastance results in a total gain of ${\sim }$2 % in maximum flow rate. The heart rate is also increased, and therefore, the ejection time is reduced by $3.550 \times 10^{-2} \, \hbox {s}$ relative to the initial supine case.

Table 5 summarizes the period averaged flow in each branch of the 3D domain. It can be seen that in the no-control case, the tilt test reduces the flow to all the upper branches. In the control case, flow is increased in all the branches of the model, despite the increase in peripheral resistance and gravity-induced increase in afterload.

### Limitations and future work

The systemic circulation was approximated as a closed-loop model containing only the left heart, large arteries, small arteries, arterioles, venules and veins. The pulmonary circulation can be added to the existing closed-loop model by including lumped parameter circuits representing the right heart and pulmonary vasculature. The addition of a more detailed model of the venous circulation, where the superior and inferior vena cavae are modeled using dedicated compartments, would have enabled a more accurate characterization of the circulating venous volume. Moreover, as changes in the heart rate and maximum elastance also affect the right heart, and therefore the venous return, the inclusion of the pulmonary circulation is a key feature which will be added in future developments of this model.

In this work, the afferent activity of the baroreceptors was modeled as a sigmoidal function of deviations in pressure. This simple model is able to describe features of the baroreceptor nervous activity such as saturation and the sigmoidal response to increasing/decreasing pressure; however, it is unable to describe other prominent features such as hysteresis, adaptation/resetting and post-excitatory depression of the nervous activity in response to changes in pressure (Mahdi et al. 2013). Future developments in our model will be enhanced to reproduce these complex features.

In this model, only the baroreceptors at the carotid bifurcation location were assumed to contribute to the baroreflex, neglecting the input from the baroreceptor cells located at the aortic arch. Future expansions of this work will include the inputs from the aortic arch baroreceptor cells, using either the local pressure or the strain computed from the fluid–structure interaction deformation field. However, such an addition is unlikely to yield significant improvements in the model as experimental studies in humans indicate that both aortic and carotid baroreceptors operate over a similar range of pressures with no significant differences in the threshold and saturation properties (Smith et al. 2001).

In this work, the change in posture was modeled by rotating the direction of the body force term in the momentum equation. This approach neglects the inertial effects of the body rotation, since the position of the body remains fixed through the simulation. In future work, a moving mesh approach will be adopted to include this feature, the effect of which is not known; as to the authors knowledge, no such simulation is reported in the blood flow literature.

The gravity field was only accounted for in the 3D domain. Here, the change in posture was not included in the lumped parameter networks representing the proximal and distal component of the closed-loop circuit. Neglecting the pressure in the 0D part of model results in an over-estimation of pressure in the upper branches (above the center of gravity given by the heart) and an under-estimation of pressure in the descending aorta (below the center of gravity given by the heart). The effect of gravity during the tilt test was previously examined in a 0D model by Heldt et al. (2002) who introduced reference pressures in the lumped parameter network that varied as a function of the orientation. Future work will include such a feature in the 0D components of the circuit.

In this work, the short-term regulation of pressure was limited to the global effects of the baroreflex. However, in the human circulation, there are localized feedback mechanisms in certain vascular beds that operate jointly with the baroreflex to regulate pressure and flow in areas such as the cerebral, coronary and renal circulations. Local changes in pressure, wall shear stress and/or metabolite concentrations trigger vascular smooth muscle activation, affecting the arteriolar resistance through changes in vessel diameter. In order to model such features in future developments of this model, it is intended to adopt localized autoregulatory functions such as those described in the 0D models developed by Carlson et al. (2008). A modeling framework with such global and local auto-regulatory blood flow mechanisms will be a stepping stone toward more ambitious modeling topics such as the simulations of trauma, surgical interventions and acute responses to stress.

**Fig. 9:**
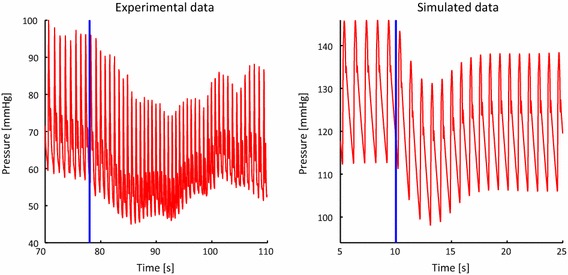
Comparison of experimental and simulated pressure recovery during the head up tilt test. The experimental data was obtained by digitizing the data contained in Williams et al. (2013) using the software Plot Digitizer http://plotdigitizer.sourceforge.net/. The simulated data is taken from the left internal carotid vessel in the control case. In both plots, the start of the tilt test is denoted with the *solid blue line*

Lastly, future work will be devoted toward validating this model against experimental tilt test data. In Fig. 9, our simulated pressure response during the tilt test is compared against the experimental data reported by Williams et al. (2013). It can be seen that the simulated response qualitatively follows the same trend as the data, showing an initial decrease in pressure followed by a gradual increase in which pre-tilt values are not fully recovered. There are, however, differences in heart rate, pressure range and duration of recovery, differences that clearly suggest that specific values of the gains and time constants must be identified on a patient-specific basis.

## Conclusions

In this study, the effects of the baroreflex were assessed in an image-derived, patient-specific, fluid–solid interaction, closed-loop model of the systemic circulation. Described using a coupled 3D–0D approach, the large arteries have been modeled in 3D, with the left heart and remaining systemic arteries and veins in 0D. Integrated into this model is the arterial baroreflex—a negative feedback system that functions to maintain global pressures throughout the circulation.

Here, the baroreflex was triggered by virtually simulating a clinical procedure known as the tilt test, in which the posture of the patient is rapidly altered—therefore triggering the response of the baroreflex. In this model, the orientation of the gravity vector is modified over time, inducing a pressure gradient throughout the model. Comparing pressures and flows throughout this closed-loop model, with and without the baroreflex control, highlights the sensitivity of the carotids to changes in orientation and the ability of the baroreflex to restore pressures globally throughout the circulation.
